# The Possible Role of the Novel Cytokines IL-35 and IL-37 in Inflammatory Bowel Disease

**DOI:** 10.1155/2014/136329

**Published:** 2014-08-18

**Authors:** Yanmei Li, Yanan Wang, Ying Liu, Yatian Wang, Xiuli Zuo, Yanqing Li, Xuefeng Lu

**Affiliations:** Department of Gastroenterology, Qilu Hospital, Shandong University, 107 Wenhua West Road, Jinan 250012, China

## Abstract

Interleukin- (IL-) 35 and IL-37 are newly discovered immune-suppressing cytokines. They have been described in inflammatory diseases such as collagen-induced arthritis and asthma. However, their expressions in inflammatory bowel disease (IBD) patients have not been yet explored. Our aim was to evaluate serum and inflamed mucosal levels in IBD patients. In 20 ulcerative colitis (UC) patients, 7 Crohn's disease (CD) patients, and 15 healthy subjects, cytokine levels in serum were determined using ELISA and mucosal expression studies were performed by immunohistochemistry, quantitative real-time PCR, and Western blot. The results showed that serums IL-35 and IL-37 levels were significantly decreased in UC and CD patients compared with healthy subjects. The cytokines levels correlated inversely with UC activity. IL-35 was expressed in infiltrating immune cells while IL-37 in intestinal epithelial cells as well as inflammatory cells. IBD patients had significantly higher *Ebi3*, *p35* (two subunits of IL-35), and *IL-37b* gene expressions; IL-35 and IL-37 protein expressions were higher in IBD patients compared with controls. The study showed that serums IL-35 and IL-37 might be potentially novel biomarkers for IBD. Intestinal IL-35 and IL-37 proteins are upregulated, suggesting that regulating the expression of the two cytokines may provide a new possible target for the treatment of IBD.

## 1. Introduction

Inflammatory bowel disease (IBD), including Crohn's disease (CD) and ulcerative colitis (UC), is a kind of chronic inflammatory disorder of the gastrointestinal tract. Although the etiology is not completely understood, initiation and exacerbation of the inflammatory process seem to be due to a massive local mucosal immune response [1]. Analysis of immunoinflammatory pathways in the gut of patients with UC or CD has shown that tissue damage is driven by a complex and dynamic crosstalk between immune and nonimmune cells and that cytokines are key mediators of this interplay [2, 3].

Interleukin- (IL-) 35 and IL-37 are newly discovered immune-suppressing cytokines. IL-35 belongs to the IL-12 family, which contains IL-12, IL-23, and IL-27. It is composed of two subunits, Epstein-Barr virus-induced gene 3 (Ebi3) and p35 (IL-12a) [4]. IL-35 was shown to be secreted by Foxp3+CD4+CD25+ regulatory T cells (Tregs) in mice or a regulatory T cell population induced by IL-35 [5] and CD138+ plasma cells in experimental autoimmune encephalomyelitis (EAE) [6]. Using experimental database mining and statistical analysis methods, Li et al. reported that IL-35 is not constitutively expressed in human tissues but it is inducible in response to inflammatory stimuli [7]. IL-37, also known as IL-1F7, is a new member of the IL-1 family, which shares common characteristic symbolized by a similar *β*-barrel structure. IL-37b is the largest isoform of the five variants and is expressed in a variety of normal tissues and tumors in humans [8]. It was first found in bone marrow, and neutrophils were the main place for its synthesis. It is mainly expressed in blood cells, respiratory tract, gastrointestinal tract, and skin keratinocytes [9].

To investigate the possible role of IL-35 and IL-37 in the inflammatory process of IBD, we aim to evaluate serum and mucosal levels in IBD patients. To the best of our knowledge, this is the first study that explores expression through a quantitative real-time polymerase chain reaction (qRT-PCR), immunohistochemistry, and Western blot of IL-35 and IL-37 in inflamed colonic mucosa of IBD patients.

## 2. Materials and Methods

### 2.1. Subjects

A total of 27 patients with definitive diagnosis of IBD were recruited at Shandong University Qilu Hospital. 20 UC and 7 CD patients were included during the period from September 2013 to April 2014. Diagnosis was performed by the presence of history of abdomen pain, diarrhea, or blood in stool and macroscopic appearance by colonoscopy or double balloon endoscopy and biopsy compatible with IBD. The following were relevant medical records: gender, age at diagnosis, disease evolution, extension, extraintestinal manifestations, medical treatment, and clinical course of disease. UC activity was assessed by Mayo score activity index [10] and CD activity was assessed by Crohn's disease activity index (CDAI) [11]. Blood was drawn for the measurement of hemoglobin, hematocrit, and erythrocyte sedimentation rate (ESR). Additionally, 15 noninflamed controls (median age, 48 yr; 9 males/6 females) were recruited among healthy subjects undergoing a colonoscopy because of screening for colorectal cancer or polyp surveillance. These subjects were free from gastrointestinal symptoms and other inflammatory diseases. Only subjects with both macroscopically and microscopically normal colonoscopy were included. None of the healthy subjects in the study was taking any medications known to affect the gastrointestinal tract or the immune system.

### 2.2. Samples Collection

A fasting blood sample was taken from all patients and healthy subjects. It was centrifuged at 1500 ×g for 20 min at room temperature and the serum was collected and stored at −80°C until analysis. During endoscopy, biopsies from colon or ileocecum or small intestine were obtained from patients and healthy subjects. Two biopsies to be used for RNA and protein assessment were snap-frozen in liquid nitrogen and then transferred to −80°C for storage until processing. One biopsy was placed in formalin for pathology and immunohistochemical staining. The study was performed after receiving written informed consent from all study subjects, and the protocol was approved by the Regional Ethical Review Board at Qilu Hospital, Shandong University.

### 2.3. Enzyme-Linked Immunosorbent Assay

Serums IL-35 and IL-37 were measured using commercially available enzyme-linked immunosorbent assay (ELISA) kits (Bio-Swamp). All cytokines assays were performed in duplicate and in accordance with the manufacturers' protocols.

### 2.4. Immunohistochemistry

Formalin-fixed and paraffin-embedded 4 *μ*m thick tissue slices were dewaxed and rehydrated before antigen retrieval. Microwave antigen retrieval method was then preformed with the slides immersed in EDTA antigen retrieval solution (ph 9.0) for 15 minutes. After that, 3% hydrogen peroxide (H_2_O_2_) was added on the slides to inhibit the endogenous peroxidase activity. Nonspecific binding was blocked by incubation with 10% normal goat serum in 37°C, pH7.5, for 30 min. Subsequently, mouse anti-human IL-35 monoclonal antibody (Imgenex, USA) at a 1 : 200 dilution and mouse anti-human IL-37 monoclonal antibody (Abcam, USA) at a 1 : 250 dilution were applied, respectively, to the sections that were latter incubated at 4°C overnight. On the second day, biotinylated antibody and streptavidin-peroxidase reagent (Zhongshan Biotech, China) were successively applied for 30 min each at 37°C. Finally, 3′-diaminobenzidine tetrahydrochloride (DAB) was used for visualization and hematoxylin was added to counterstain. Samples were viewed with Olympus IX81 microscope and images were produced using DP Controller 1.2.1.108. All of the slides were independently analyzed by two pathologists.

### 2.5. RNA Isolation and Quantitative Real-Time PCR

RNA was extracted using Trizol Reagent (Takara, Japan), following the manufacturer's guidelines. First-strand cDNA was synthesized by Realtime PCR Master Mix Kit (TOYOBO, Japan) in a volume of 10 *μ*L. For quantitative real-time PCR (qRT-PCR), the LightCycler 4.0 instrument (Roche Applied Science, Germany) and the SYBR  Green Realtime PCR Master Mix Kit (TOYOBO, Japan) were used according to the protocol provided by the manufacturer. The following primers were used:* Ebi3*: forward 5′-GCA GCA GAC GCC AAC GT-3′, reverse 5′-CCA TGG AGA ACA GCT GGA CAT-3′;* p35*: forward 5′-CCT TCA CCA CTC CCA AAA C-3′, reverse 5′-TGT CTG GCC TTC TGG AGC AT-3′.* IL-37*: forward 5′-GCT CAG GTG GGC TCC TGG AA-3′, reverse 5′-GCT GAC CTC ACT GGG GCT CA-3′. Human glyceraldehyde-3-phosphate dehydrogenase (*GAPDH*) was used as internal control from parallel samples because the reference gene was stably expressed, and its primers were forward: 5′-GGT GGT CTC CTC TGA CTT CAA CAG-3′ and reverse: 5′-GTT GTT GTA GCC AAA TTC GTT GT-3′. Melting curve analysis was used to confirm amplification specificity. The quantification data were analyzed with LightCycler analysis software version 4.0 (Roche Applied Science, Germany) and the relative target gene expression was normalized on the basis of* GAPDH*. Results were expressed as an x-fold difference relative to the calibrator.

### 2.6. Western Blot Analysis

Frozen tissue samples were lysed in RIPA buffer (50 mM Tris, pH 7.4, 150 mM NaCl, 1% Triton X-100, 1% sodium, deoxycholate, 0.1% SDS; Beyotime, China) and PMSF (Beyotime, China) followed by centrifugation (12,000 rpm, 4°C, 20 minutes), after which the supernatants were stored at −80°C until use. The protein concentrations of the lysates were determined using an enhanced BCA Protein Assay Kit (Beyotime, China). Extracted proteins were subjected to sodium dodecyl sulfate-polyacrylamide gel electrophoresis (SDS-PAGE). Samples transferred onto PVDF membranes were treated with a 1 : 200 dilution of mouse anti-human IL-35 monoclonal antibody (Imgenex, USA) and anti-human IL-37 monoclonal antibody (Abcam, USA) followed by a 1 : 5,000 dilution of sheep anti-mouse horseradish peroxidase-conjugated secondary antibodies (Zhongshan Biotech, China). As a control, rabbit anti-human GAPDH polyclonal antibody (Proteintech, USA) and peroxidase-conjugated anti-rabbit IgG (H+L) (Proteintech, USA) were used to normalize. Immunoreactivity was visualized with the Immobilon Western Chemiluminescent HRP Substrate (Millipore, USA) and quantified using Image J software (http://rsbweb.nih.gov/ij/index.html).

### 2.7. Statistical Analysis

Statistical evaluations were performed with GraphPad Prism 5.0 (GraphPad Software Inc., San Diego, CA, USA) and SPSS19.0 (SPSS Inc., Chicago, IL, USA). Data were expressed as mean ± standard error (SEM). The Mann-Whitney test was used to evaluate differences of cytokine expression in the serum. One-way analysis of variance, followed by post hoc *t* tests with Newman-Keuls test for multiple comparisons, was used to compare the 3 groups. Pearson correlation was used to calculate correlations between serum cytokines levels and Mayo score. One-way analysis of variance, followed by post hoc *t* tests with Tukey correction for multiple comparisons, was used to compare the 3 groups in differences of mucosal expression. In all tests, a *P* value < 0.05 was considered significant.

## 3. Results

### 3.1. Demographic and Clinical Characteristics of IBD Patients

They are described in Table 1. The Montreal classification was used to define the extent of UC: 50% had pancolitis (E3), 35% had left-sided colitis (E2), and 15% had proctitis (E1). According to Mayo score, six UC patients (30%) showed mild activity, seven (35%) moderate activity, and seven (35%) severe activity. Six UC patients (20%) had extraintestinal manifestations, including arthropathy (15%), primary sclerosing cholangitis (10%), and erythema nodosum (5%). All patients were under mesalazine treatment; 45% used oral or systemic glucocorticosteroids; 5% were taking azathioprine. CD patients were few. Five were moderate activity and two were severe activity. Two patients had arthropathy.

### 3.2. Serums IL-35 and IL-37 Levels Are Decreased in IBD Patients

**Figure 1:**
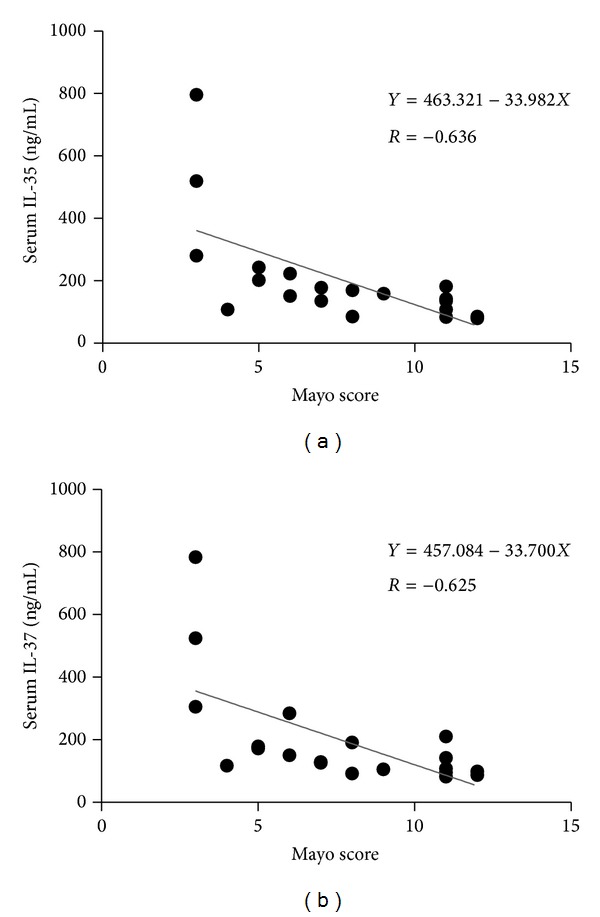
There was an intermediate inverse correlation between the Mayo score and serum IL-35 levels (a). IL-37 levels were moderately negatively correlated with Mayo score (b).

Serums IL-35 and IL-37 concentrations were significantly reduced in the active UC patients and active CD patients compared with healthy controls (HC) (Table 2(a)). There were also significant differences between mild UC and moderate UC and mild UC and severe UC (*P* < 0.05) (Table 2(b)). In contrast, UC and CD group and moderate and mild UC group seem to be statistically meaningless. We also assessed whether the serum cytokine levels were associated with the Mayo score in UC patients. The results showed that lower IL-35 and IL-37 levels were moderately negatively correlated Mayo score. (*R* = −0.636, *P* < 0.05; *R* = −0.625, *P* < 0.05, resp.) (Figures 1(a) and 1(b)).

### 3.3. IL-35 Expression in Colonic Mucosa from IBD Patients

**Figure 2:**
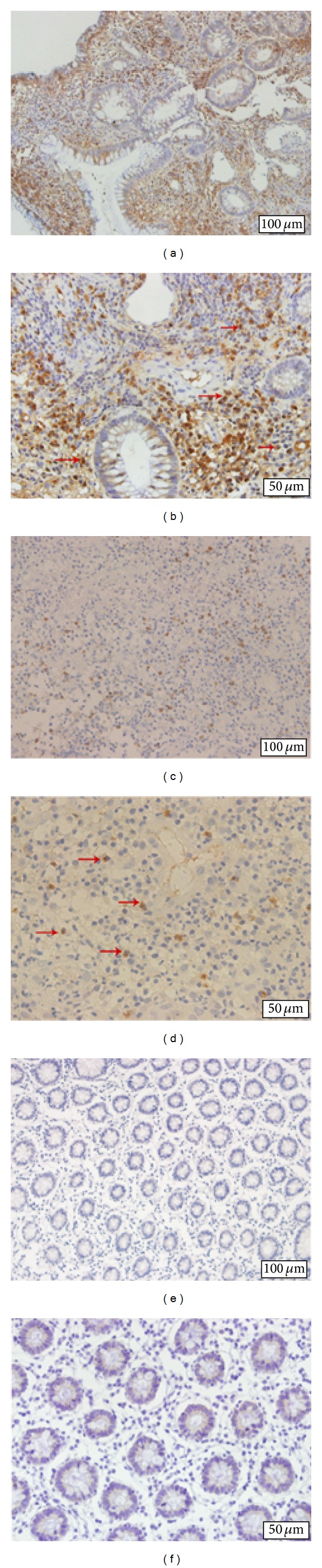
Photomicrographs of immunostaining for IL-35 in human colon from patients with UC (a and b), patients with CD (c and d), and healthy controls (e and f). No staining was found in the control group (e and f), and IL-35 was expressed in infiltrating immune cells (morphologically resembling lymphocytes) but not in epithelial cells. The staining was mostly cytoplasmic, and red arrows depicted immunoreactive positive cells. The number of IL-35-expressing cells in inflamed colonic tissue of patients with UC was higher than in CD patients' tissue. Original magnification: (a), (c), (e) ×200; (b), (d), (f) ×400.

**Figure 3:**
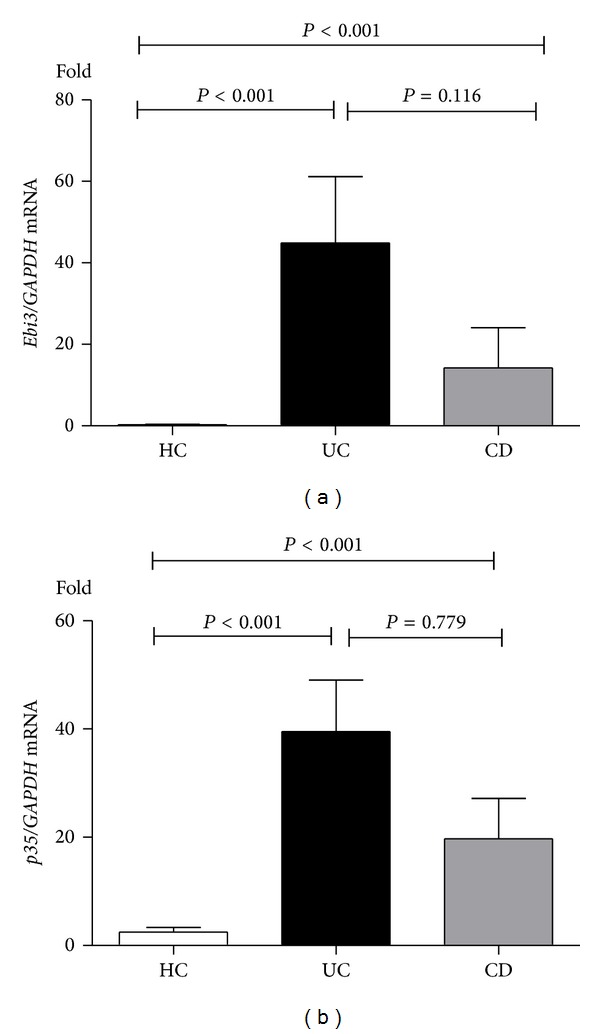
Detection of* Ebi3* and* p35* mRNA by qRT-PCR.* Ebi3* (a) and* p35* (b) were significantly overexpressed in endoscopic specimens in both UC patients and CD patients as compared to controls (*P* < 0.001).* Ebi3* and* p35* mRNA expression in UC and CD biopsies did not reach statistical significance.

**Figure 4:**
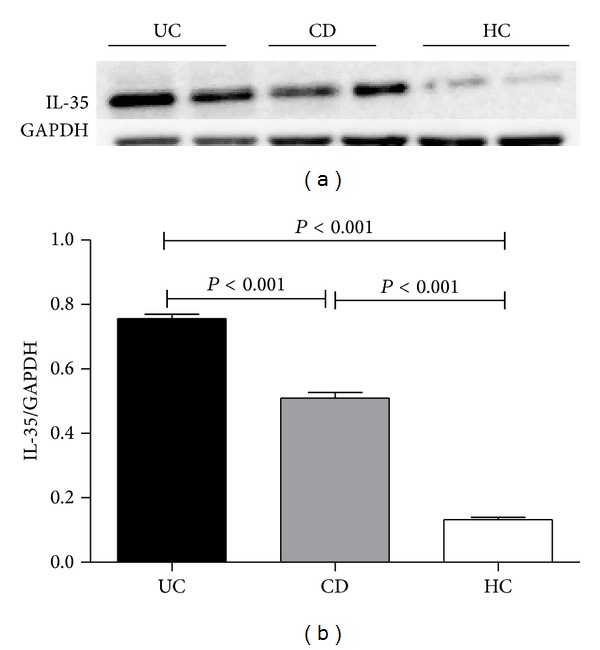
Detection of IL-35 in protein extracts of endoscopic specimens by Western blot. Representative Western blot of IL-35 and GAPDH (a) proteins levels in UC and CD patients and healthy controls. A significant upregulation of IL-35 was observed in inflamed mucosa of patients as compared to controls (*P* < 0.001) and IL-35 protein expression in UC biopsies was higher than that in CD biopsies (b).

In order to determine gene and protein expressions in UC and CD patients, mRNA relative expressions of* Ebi3* and* p35* and protein of IL-35 were quantified by qRT-PCR and Western blot analysis. IL-35 producing cells were determined by immunohistochemistry. As shown in the representative images of this analysis in Figures 2(a)–2(d), IL-35 was expressed in infiltrating immune cells but not in epithelial cells. Normal colon tissue from healthy subjects had no IL-35 expression at all (Figures 2(e) and 2(f)). IL-35 positive cells were localized mainly in inflammatory infiltrates, predominantly mononuclear cell (lymphocytes). The number of IL-35-expressing cells in inflamed colonic tissue of patients with UC (Figures 2(a) and 2(b)) was higher than that in CD patients' tissue (Figures 2(c) and 2(d)). IBD patients had significantly higher* Ebi3* and* p35* gene expression compared with healthy control group (*P* < 0.0001) (Figure 3). And the difference between UC and CD biopsies in* Ebi3* and* p35* mRNA expressions did not reach statistical significance (*P* = 0.116; *P* = 0.779). Western blot analysis showed a significant upregulation of IL-35 (*P* < 0.05) in inflamed mucosa of patients as compared to controls (Figure 4). IL-35 protein expression in UC biopsies was found higher than that in CD biopsies. No relationship between IL-35 mucosal expression and the activity was observed in UC and CD patients.

### 3.4. IL-37 Expression in Colonic Mucosa from IBD Patients

**Figure 5:**
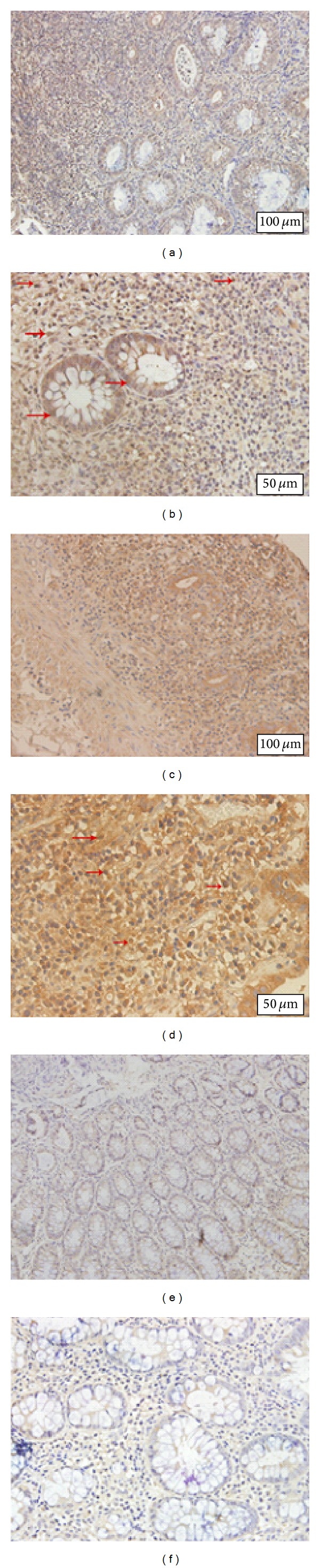
Photomicrographs of immunostaining for IL-37 in patients with UC (a and b), patients with CD (c and d), and control subjects (e and f). IL-37 was expressed by both immune and epithelial cells (a–d), and normal tissue had IL-37 expression, though with relatively small amount (e and f). Besides strong cytoplasmic staining, few single lamina propria mononuclear cells showed nuclear expression of IL-37.

**Figure 6:**
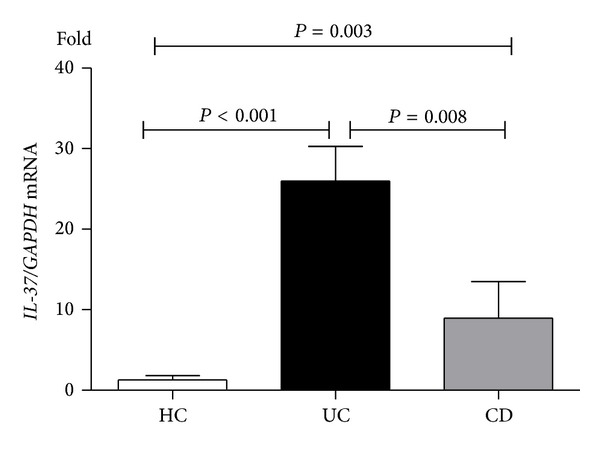
*IL-37* gene expression in colonic mucosa from IBD patients. A significant increase in* IL-37* mRNA was shown in inflamed mucosa of patients as compared to controls.

**Figure 7:**
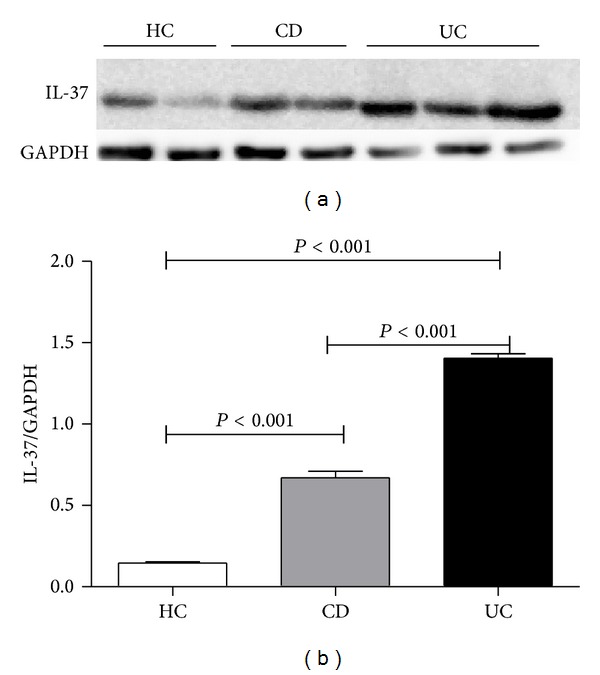
Detection of IL-37 in protein extracts of endoscopic specimens by Western blot. Representative Western blot of IL-37 and GAPDH (a) proteins levels in UC and CD patients and healthy controls. IL-37 protein expression was found to be higher in UC patients and CD patients compared to healthy subjects and the expression levels of IL-37 protein were higher in the samples of UC patients than that of CD samples (b).

Immunohistochemistry showed that both immune and epithelial cells could express IL-37  (Figures 5(a)–5(d)), and normal tissue had IL-37 expression, though with relatively small amount (Figures 5(e) and 5(f)). Besides strong cytoplasmic staining, few single lamina propria mononuclear cells show nuclear expression of IL-37. Similarly, compared with control group, IBD group had higher* IL-37* mRNA expression (*P* < 0.001), whereas difference between UC and CD was still statistically significant (*P* < 0.001) (Figure 6). For IL-37, protein expression trend runs like IL-35. Western blot showed that IL-37 protein expression was found to be higher in UC patients and CD patients compared to healthy subjects and the expression levels of IL-37 protein were higher in the samples of UC patients than that of CD samples (*P* < 0.001) (Figure 7). The mean IL-37 expression tended to be higher in severe UC samples than in mild UC samples but was not statistically significant.

## 4. Discussion

In this study, we first demonstrated that serums IL-35 and IL-37 levels were significantly lower in active IBD patients than healthy controls and were moderately negatively correlated with Mayo score in UC patients. Takahashi et al. reported that the Treg cell frequency decreased in the active stage of UC and it correlated inversely with the disease activity [12]. Their results suggested that a deficiency of Treg cells was associated with the progression of ulcerative colitis. Treg cells are the main source of IL-35, which may result in the reduction of IL-35 in peripheral blood. Through immunocytochemical staining, IL-37 protein is present mainly in the cytoplasm of peripheral blood mononuclear cells (PBMC) and constitutively at low levels in normal people and can be upregulated by inflammatory stimuli and cytokines [13]. IL-37 is expected to have the function of translocation into nucleus [14] and can be redistributed between intracellular and extracellular. Perhaps IL-37 transferring to intracellular is the reason why the content is down in serum. The study shows that decreased serums IL-35 and IL-37 levels may represent insufficient anti-inflammatory activity in vivo and hold promise as novel biomarkers for monitoring disease activity in UC.

We have characterized the mucosal expressions of IL-35 and IL-37 in patients with IBD. The overexpression and enhanced production of IL-35 and IL-37 in colonic mucosa may play a role in the inflammatory process of IBD. Treg cells are highly infiltrated in the lamina propria (LP) of inflamed areas of UC colon compared to normal colon [15]. Treg cells induced the generation of induced regulatory T 35 cells (iTR 35 cells) in an IL-35- and IL-10-dependent manner in vitro and induced their generation in vivo under inflammatory conditions in intestines infected with* Trichuris muris*. So we think iTR 35 cells are increased to produce more IL-35 to inhibit effective T cell (Teff cell) proliferation and suppress Th17 development. Maybe the IL-35-producing B cells also participate the production of IL-35, for Shen et al. suggested that, during experimental autoimmune encephalomyelitis (EAE), CD138 (+) plasma cells were also the main source of B-cell-derived IL-35 and IL-10 [6]. In the gut, constitutive epithelial expression of anti-inflammatory immune mediators like IL-37 might be mandatory to maintain the homeostasis of the local immune response against commensal bacteria. The protein is induced by toll-like receptor (TLR) agonists in monocytes and is expressed in tissues from patients with inflammatory diseases [16, 17]. And the production of IL-37 by epithelial cells, neutrophils, and monocytes can form a positive feedback to promote more. We speculate that the increased IL-35 and IL-37, which are delivered by infiltrating immune cells, counteract mucosal inflammation in IBD. The UC group possessed the highest expression of IL-35 and IL-37 in colonic tissue, followed by CD group, and HC group expressed the lowest. The lower anti-inflammatory cytokines in CD may explain why CD is a more chronic and continued disease. However, there was no relationship between IL-35 and IL-37 mucosal expressions and the activity in UC or CD patients. It is possible that, at the beginning of inflammatory disease, a large number of the immune-suppressing cytokines were stimulated to produce to limit inflammation in the affected colon. Despite their increased frequency and potent suppressor activity in vitro, they fail to reverse the disease process. Unlike* IL-37*, the mRNA levels of* Ebi3* and* p35* did not show differences between UC and CD. We should consider the fact that IL-27 and IL-35 shared the *β*-chain Ebi3 whereas IL-12 and IL-35 shared the p35 a-chain.

The mechanisms of IL-35 and IL-37 are not clear until now. IL-35 is involved in inflammatory diseases in the nervous system, alimentary system, bone and joint system, and respiratory system. Zandian et al. demonstrated that IL-35 had an inhibitory effect against demyelination by preventing the development of autoaggressive T cells [18]. Kochetkova et al. suggested that exogenous IL-35 could suppress the activity of CD4+T cells, and Th1 and Th17 cells and inhibit the inflammation of collagen-induced arthritis [19]. Meanwhile, it was indicated that IL-35 could help the respiratory system recover from inflammation [20]. Wirtz et al. recently confirmed that IL-35 could significantly suppress Th1 and Th17 cells' proliferation, reduce the development of experimental colitis, and protect the intestine from immune responses in mice [21]. One subunit of IL-35, Ebi3, is widely expressed in EBV-transformed B-lymphocytes and tissues, such as tonsil and spleen [22]. Ebi3 could negatively regulate IL-17, IL-22, and Th17 transcription factor ROR*γ*t and exert protective immunity against inflammation [23]. The subunit of IL-12, p35, could lead to the progression of Herpes stromal keratitis (HSK) in mice, which is IL-12p40 independent [24]. The two subunits of IL-35 do have their own ability to regulate immunity and the process of inflammation. When they combine together to form the heterodimer, the p35 subunit may act as a ligand, and the other subunit EBI3 may mainly exert its immunological function [25]. So far, the signaling pathway of IL-35 is not clear yet. Meanwhile, the research confirmed that IL-35 signaled through a unique heterodimer of receptor chains IL12R*β*2 and gp130 or homodimers of each chain [26]. Signaling through the IL-35 receptor required the transcription factors STAT1 and STAT4, which formed a unique heterodimer that bounded to distinct sites in the promoters of the genes encoding the IL-12 subunits p35 and Ebi3. IL-35 can directly suppress Teff cell proliferation, convert naive T cells into IL-35-producing iTr35 cells, suppress Th17 development, and mediate IL-10 generation. Similarly, IL-37 is a cytokine for inflammation, autoimmunity, and other immunological disorders. The IL-37 protein is highly expressed in synovial cells of patients with rheumatoid arthritis but expressed at low levels in healthy human synovial cells [5, 27]. IL-37 expression was also significantly increased in the skin lesions of patients with psoriasis and in macrophages of Crohn's disease lesions [28]. IL-37 is synthesized as a proprotein which, after stimulation, is processed to its mature form [28]. Lipopolysaccharide (LPS), together with other inflammatory stimuli and cytokines, activates caspase-1 and is considered to be the major cleaving enzyme responsible for maturation of IL-1 family precursors [16]. With broad-spectrum function in antibacterial, antiviral, neutralizing endotoxins and antitumor and immune regulation, IL-37 can kill the microorganism in general. And its mechanism is mainly by changing the permeability of bacterial cells. It also has the ability to raise the production of several cytokines such as IL-8 to expand acquired immune function [29]. Studies of mouse models have reached the result that IL-37 downregulates inflammation [3]. TLR, tumour necrosis factor (TNF), and other cytokines can induce the production of inflammatory cytokines. Nold et al. reported that IL-37 attenuated the abovementioned process, thus exerting anti-inflammatory effects [27]. Besides, Liu et al. proved that IL-37 exerted a significant inhibition on TNF-*α*-induced IP-10 expression [30]. In the inflamed mucosa of IBD patients, T cell activation, as well as dendritic cells (DCs) activation, can be inhibited by epithelial cell-derived IL-37. The possible mechanism may be that IL-37 reduces the surface expressions of the costimulatory molecule CD86 (B7-2) and major histocompatibility complex (MHC) II on DCs. IL-37b mRNA expression induced by TNF-*α* was mediated by the activation of MAPK and PI3K and transcription factors NF-kB and AP-1. Conversely, these signalling molecules are major mediators of the induction of proinflammatory responses in the inflamed mucosa. Thus, it became clear that in the inflamed mucosa of IBD patients a negative feedback inhibitor of inflammatory responses (the induction of IL37b) and proinflammatory responses was induced via coupled signalling pathways [31].

## 5. Conclusion

IL-35 and IL-37 are brand new potential therapeutic cytokines for IBD. Our experiment group (namely, UC group and CD group) includes 27 cases, and large-scale testing needs to be performed. Thus, further mechanism studies on the roles of IL-35 and IL-37 should be performed to make it available and useful in the future.

## Figures and Tables

**Table 1:** Demographic and clinical characteristics of IBD patients.

Patients number	Sex	Age at	Disease	Disease extent	Disease activity	Current therapy
		diagnosis	duration			
		(years)	(months)			
					Mayo score	

UC1	F	46	4	E2	7	Mesalazine
UC2	M	29	70	E3	12	Mesalazine, corticosteroids
UC3	M	25	2	E3	9	Mesalazine, corticosteroids
UC4	F	51	10	E3	11	Mesalazine, corticosteroids
UC5	M	57	12	E1	5	Mesalazine,
UC6	M	34	6	E2	6	Mesalazine,
UC7	F	28	168	E2	3	Mesalazine,
UC8	M	34	3	E3	11	Mesalazine, corticosteroids
UC9	F	28	40	E2	11	Mesalazine, corticosteroids, and AZA
UC10	M	59	7	E3	11	Mesalazine, corticosteroids
UC11	M	24	1	E3	8	Mesalazine, corticosteroids
UC12	F	27	1	E1	4	Mesalazine, enema
UC13	M	27	1	E1	3	Mesalazine
UC14	M	58	2	E2	12	Mesalazine, corticosteroids
UC15	M	65	2	E2	5	Mesalazine
UC16	F	15	2	E2	11	Mesalazine, corticosteroids
UC17	M	22	12	E3	6	Mesalazine
UC18	M	37	1	E3	3	Mesalazine
UC19	F	63	1	E3	8	Mesalazine
UC20	F	28	5	E3	7	Mesalazine, enema
Average (SEM)		**37.85 (3.48)**	**17.50 (8.76)**		**7.65 (0.72)**	

					CDAI score	

CD1	M	60	2	L1	7	Mesalazine, corticosteroids, and AZA
CD2	M	39	180	L1	6	Mesalazine, AZA
CD3	M	28	5	L2	8	Mesalazine, corticosteroids
CD4	F	26	1	L3	9	Mesalazine, corticosteroids
CD5	M	49	60	L4	9	Mesalazine, corticosteroids
CD6	M	50	3	L3	6	Mesalazine
CD7	M	22	6	L1	7	Mesalazine, corticosteroids
Average (SEM)		**39.15 (5.44)**	**36.71 (25.18)**		**7.43 (0.48)**	

UC: ulcerative colitis; CD: Crohn's disease; E1: proctitis; E2: leftsided colitis; E3: pancolitis; L1: ileal; L2: colonic; L3: ileocolonic; L4: upper GI involvement; AZA: azathioprine.

**(a):** 

	UC (*n* = 20)	CD (*n* = 7)	HC (*n* = 15)
Il-35 level (ng/mL)	203.36 ± 38.21∗∗	454.17 ± 219.38∗∗	1788.96 ± 209.43
Il-37 level (ng/mL)	199.28 ± 38.60∗∗	481.67 ± 232.82∗∗	2275.68 ± 261.24

**(b):** 

	Mild UC (Mayo 3~5) (*n* = 6)	Moderate UC (Mayo 6~10) (*n* = 7)	Severe UC (Mayo 11~12) (*n* = 7)
Il-35 level (ng/mL)	358.26 ± 103.95^∗#^	157.29 ± 15.89	116.69 ± 14.48
Il-37 level (ng/mL)	346.97 ± 105.83^∗#^	154.21 ± 24.95	117.75 ± 17.14

**P < 0.001 versus HC; *P < 0.05 versus mild UC; ^#^ *P* < 0.05 versus severe UC.

## Abbreviations

IL-35: Interleukin 35
IL-37: Interleukin 37
CD: Crohn's disease
UC: Ulcerative colitis
IBD: Inflammatory bowel disease
PBMC: Peripheral blood mononuclear cells
qRT-PCR: Quantitative real-time polymerase-chain reaction
iTR35: Induced regulatory 35 cell
Teff: Effective T cell
TLR: Toll-like receptor
DCs: Dendritic cells.
